# Spatiotemporal Regulation of *Vibrio* Exotoxins by HlyU and Other Transcriptional Regulators

**DOI:** 10.3390/toxins12090544

**Published:** 2020-08-22

**Authors:** Byoung Sik Kim

**Affiliations:** Department of Food Science and Engineering, ELTEC College of Engineering, Ewha Womans University, Seoul 03760, Korea; b.kim@ewha.ac.kr

**Keywords:** exotoxin, transcriptional regulation, hemolysin, cytolysin, MARTX toxin, secreted phospholipase, HlyU, *Vibrio* species

## Abstract

After invading a host, bacterial pathogens secrete diverse protein toxins to disrupt host defense systems. To ensure successful infection, however, pathogens must precisely regulate the expression of those exotoxins because uncontrolled toxin production squanders energy. Furthermore, inappropriate toxin secretion can trigger host immune responses that are detrimental to the invading pathogens. Therefore, bacterial pathogens use diverse transcriptional regulators to accurately regulate multiple exotoxin genes based on spatiotemporal conditions. This review covers three major exotoxins in pathogenic *Vibrio* species and their transcriptional regulation systems. When *Vibrio* encounters a host, genes encoding cytolysin/hemolysin, multifunctional-autoprocessing repeats-in-toxin (MARTX) toxin, and secreted phospholipases are coordinately regulated by the transcriptional regulator HlyU. At the same time, however, they are distinctly controlled by a variety of other transcriptional regulators. How this coordinated but distinct regulation of exotoxins makes *Vibrio* species successful pathogens? In addition, anti-virulence strategies that target the coordinating master regulator HlyU and related future research directions are discussed.

## 1. Introduction

The genus *Vibrio* is composed of various bacterial species that are metabolically versatile. They are commonly found in a wide range of marine environments [1,2]. Although some live as commensals or symbionts in crustaceans, shellfish, squid, or fish, several pathogenic species cause infectious diseases in marine animals. For instance, *Vibrio harveyi* causes luminescent vibriosis in shrimp, *V. alginolyticus* causes canker in croakers, *V. vulnificus* causes hemorrhagic disease in aquaculture eels, *V. crassostreae* causes hemocyte lysis in oysters, and *V. anguillarum* causes erythema and necrotic lesions in Pacific and Atlantic salmon [3,4,5,6,7,8].

More important, certain *Vibrio* species are human pathogens that cause various diseases in immune-compromised individuals [9]. People who consume raw or undercooked seafood can get infected by *Vibrio* species via the intestinal tract. Another route for *Vibrio* infection is an open wound submersed in contaminated seawater. Depending on the invading species and the health status of patients, symptoms can range from abdominal pain or diarrhea to bacteremia, sepsis, or even death. Well-known examples of human-infecting *Vibrio* species include *V. cholerae, V. vulnificus, V. parahaemolyticus*, and *V. alginolyticus* [9].

*Vibrio* species cause disease in humans and animals by producing diverse virulence factors. Some virulence factors promote the proliferation of invading *Vibrio* in the host and protect them from the host defense systems. For example, *V. cholerae* expresses a toxin-coregulated pilus that enables the pathogens to aggregate and adhere to gut epithelial cells, facilitating microcolony formation for initial colonization [10,11,12]. *V. vulnificus* has evolved many iron scavenging systems and shows extraordinarily rapid growth in high iron sera [13]. Indeed, multiple genes encoding biosynthetic enzymes, transporters, and utilization components for siderophores have been characterized as important virulence genes in this pathogen [14,15,16,17]. Capsular polysaccharide is another essential virulence factor for *V. vulnificus* because the encapsulated strain can resist macrophage-mediated phagocytosis [18,19]. The ability to use host-specific carbohydrates is another crucial trait for pathogens because invading bacteria compete with the host and residual commensals for nutrients [20]. Consistent with this, *Vibrio* mutants that are unable to use sialic acids, a distal end carbohydrate found in host mucins, showed colonization defects in mouse infection experiments [21,22]. Reduced virulence was also observed in *V. vulnificus* when a specific transcriptional regulator for the sialic acid utilization system was mutated [23,24]. This latter result emphasizes that virulence factors must be accurately regulated at the transcription level to enable successful pathogenesis.

**Figure 1:**
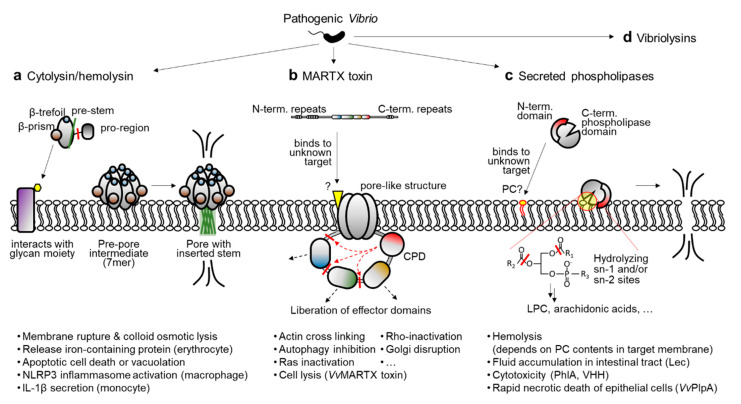
Major exotoxins produced by pathogenic *Vibrio* species. (**a**) After secretion from the bacterium, cytolysin/hemolysin is activated by processing of the pro-region. The resulting active toxin then binds to the target cell membrane, and seven monomers are assembled to form a pre-pore intermediate. During this assembly, pre-stems are extended, tightly gathered, and inserted into the membrane, establishing a β-barrel pore. (**b**) Multifunctional-autoprocessing repeats-in-toxin (MARTX) toxin secreted via atypical T1SS binds to the host cell membrane, probably by interacting with yet unidentified target molecule(s). Amino- and carboxyl-terminal repeat–containing regions of the toxin form a pore-like structure on the membrane. Central effector domains are then translocated into the host cell through that pore. In the cytosol, the cysteine protease domain (CPD) processes and liberates the cytopathic effectors. (**c**) Phospholipase is secreted from the bacterium via T2SS. At the target cell membrane, the toxin hydrolyzes the sn-1 and sn-2 sites of phospholipid substrates, producing diverse signaling molecules such as lysophosphatidylcholine (LPC). Because the toxin exhibits hemolysis activity that depends on the phosphatidylcholine (PC) content, it is speculated that the amino-terminal domain of the toxin interacts with PC on the membrane. (**d**) Proteolytic exotoxins termed vibriolysins are also produced by pathogenic *Vibrio* species. For recent reviews see [44,45,46]. The consequences of each exotoxin are summarized at the bottom of the figure. *Vv*MARTX toxin, *V. vulnificus* MARTX toxin; Lec, *V. cholerae* lecithinase; PhlA, *V. mimicus* phospholipase A; VHH, *V. harveyi* hemolysin; *Vv*PlpA, *V. vulnificus* phospholipase A.

Another kind of virulence factor in bacterial pathogens is secreted protein toxins, or exotoxins. Unlike the above-mentioned virulence factors, exotoxins are inherently destructive and affect the integrity of host cells and tissues through their enzymatic functions [25,26]. In pathogenic *Vibrio* species, especially *V. cholerae, V. vulnificus*, and *V. anguillarum*, the following exotoxins and their transcriptional regulation mechanisms have been substantially characterized: cytolysin/hemolysin, multifunctional-autoprocessing repeats-in-toxin (MARTX) toxin, secreted phospholipase, and vibriolysins (Figure 1a–d) [27,28,29,30,31,32,33,34,35,36,37,38,39]. Because the inactivation of any of these toxins in *V. vulnificus* significantly compromises its virulence [36,40,41,42], it is speculated that homologous exotoxins in other pathogenic *Vibrio* species could contribute to their virulence and disease progression. Notably, the transcriptional regulator HlyU has been found to bind to the regulatory region of all these exotoxin genes and control their expression, except for the vibriolysin genes [32,33,35,36,43]. Diverse transcriptional regulators other than HlyU also control exotoxin expression by conveying environmental cues.

It should be noted here that the details of function, pathogenic property, and transcriptional regulation mechanism of the vibriolysins have already been reviewed in other recent articles [44,45,46]. Therefore, this review will focus only on the HlyU-regulated *Vibrio* exotoxins. Accordingly, following sections summarize the functional characteristics of the three remaining *Vibrio* exotoxins, along with their regulation mechanisms governed by HlyU and other transcriptional regulators. In addition, recent attempts to develop an anti-virulence strategy by targeting HlyU will be discussed, and suggestions for future research directions will be made.

## 2. Major Exotoxins Produced by Pathogenic *Vibrio* Species

### 2.1. Cytolysin/Hemolysin

Among the various exotoxins produced by pathogenic *Vibrio* species, hemolysin has been studied extensively because it is widely distributed in vibrios and exhibits prominent outcomes as a potent exotoxin [47,48]. Hemolysin lyses the membrane of erythrocytes and releases iron-containing proteins such as hemoglobin. This is important for pathogenic *Vibrio* species because iron is a critical metal for bacterial cell proliferation, especially in a host where readily available free iron is quite limited [49,50]. Accordingly, hemolysin is considered to be a crucial virulence factor of pathogenic vibrios. 

Mainly two hemolysin families are present in *Vibrio* species [48]. One is the thermostable direct hemolysin (TDH) family, originally characterized in *V. parahaemolyticus* [51,52]. As suggested by its name, this hemolysin can withstand high temperature, and thus heating at 100 °C is not enough for toxin inactivation. The β-hemolysis activity of this TDH toxin has been linked to clinical isolates of *V. parahaemolyticus*. The THD-related hemolysins (TRHs) also exist in some *V. parahaemolyticus* strains [53]. Unlike the TDHs, however, TRHs are heat-labile and easily inactivated by heat. 

The other hemolysin family in *Vibrio* species is the El Tor hemolysin (HlyA) family, originally isolated in *V. cholerae* [48,54]. Many cell types other than erythrocytes have been reported to be affected by the HlyA family hemolysins. For instance, hemolysin from *V. cholerae* can induce apoptotic cell death in intestinal epithelial cells [55]. Furthermore, fluid accumulation and intestinal tissue damage have been linked to this family of toxins in *V. cholerae*–inoculated rabbit ileal loops and *V. vulnificus*–infected mice [40,55]. Therefore, HlyA family hemolysins in pathogenic *Vibrio* species are also called cytolysins; in *V. cholerae*, HlyA is the same toxin as *V. cholerae* cytolysin (VCC) [56]; in *V. vulnificus*, the same family of cytolysin/hemolysin is called *V. vulnificus* hemolysin (VVH) [57]; in *V. anguillarum*, it is called VAH [58]; and in *V. mimicus*, it is called VMH [59].

These cytolysin/hemolysin executes its function by forming pores in the plasma membrane of the target cells. Indeed, HlyA belongs to the β-barrel pore-forming toxin family. This family also includes other bacterial hemolysins such as aerolysin from *Aeromonas* and the β-toxin of *Clostridium* [60,61,62]. Notably, cytolysin/hemolysin in vibrios is produced as an inactive precursor containing both a signal peptide sequence and a pro-region at the amino-terminus [63]. The signal peptide is removed during translocation of the toxin from the bacterial cytosol to the periplasm, whereas the pro-region is processed by multiple proteases after the inactive pro-toxin is secreted extracellularly [64,65]. It should be noted that, unlike VCC or VMH, VVH has no pro-region [31,57]. Instead, the VVH-encoding *vvhA* gene is co-expressed with its upstream gene *vvhB*, which encodes a putative molecular chaperon for VVH that is similar to the pro-region of VCC or VMH [66]. 

In any case, the processed toxin monomers must be clustered and made into a heptameric state to form a pore in the host cell membrane (Figure 1a). First, the toxin monomer binds to the sugar molecules presented on the membrane, which is mediated by lectin-like domains at the carboxyl-terminal region of the toxin. For instance, recent studies have revealed that a β-prism domain of VCC tightly interacts with a branched pentasaccharide moiety (GlcNAc_2_-Man_3_) typically found in the complex *N*-glycans of mammalian cell-surface proteins [67,68]. After binding to the membrane, monomeric toxins are self-assembled into a heptamer and form a pre-pore intermediate [69]. During this transition, the pre-stem loop of each protomer detaches from the core part of the protein and forms an anti-parallel β-hairpin stem. The hairpins extracted from seven protomers are then tightly assembled and inserted into the membrane to form a β-barrel pore (Figure 1a) [61,69].

The consequence of pore formation by cytolysin/hemolysin is not just membrane rupture and the colloid osmotic lysis of target cells. As reviewed by Khilwani and Chattopadhyay, VCC can induce apoptotic cell death signaling and vacuolation in various types of epithelial cells [70]. In case of immune cells, cytolysin/hemolysin can induce inflammatory responses. For example, VCC and VVH activate the NOD-like receptor family pyrin domain-containing 3 (NLRP3) inflammasome in murine macrophages [71]. Similarly, VCC has been shown to cause NLRP3 and ASC (apoptosis-associated speck-like protein-containing a CARD domain)-dependent secretion of interleukin-1β from human THP-1 monocytes [72]. In combination with the lysis of iron-containing erythrocytes, these pro-inflammatory consequences of cytolysin/hemolysin emphasize the importance of its precise regulation in pathogenic *Vibrio* species.

### 2.2. MARTX Toxin

A study aiming to identify the reasons for the remaining reactogenicity in live attenuated *V. cholerae* vaccine strains identified a gene cluster that corresponds with cytotoxic activity toward Hep-2 cells [73]. A sequence analysis revealed that the gene cluster is physically linked to the cholera toxin (CTX) prophage genes in chromosome, but it encodes components of the RTX (repeats in toxin) family toxin system: RTX toxin, toxin activator, and toxin secretion proteins [73]. Similar to other RTX family toxins in pathogenic bacteria, *V. cholerae* RTX toxin has glycine/aspartic acid (GD)-rich repeats and a secretion signal peptide sequence at the carboxyl-terminal region. However, unlike the prominent RTX toxin from pathogenic *Escherichia coli*, the *V. cholerae* RTX did not show any apparent hemolytic activity. Instead, other cytopathic activities, such as actin-cross linking activity and Rho protein-inactivation, were observed. These activities have been linked to two distinct domains of the toxin [74,75,76]. 

Later, other putative cytotoxic/cytopathic effector domains were further found in this *V. cholerae* RTX toxin and its homologous toxins in other pathogenic bacteria [77,78]. Notably, a cysteine protease domain (CPD) responsible for the effector domain processing is also present in the toxins [77,79]. Because cytotoxic/cytopathic effector domains are delivered into the host cells along with the CPD, and the CPD is activated by the host specific molecule inositol hexakisphosphate, the effector domains are thought to be liberated from the holo-RTX toxin in the host cells (Figure 1b) [77,80]. To acknowledge the structural and functional features of the *V. cholerae* RTX toxin and its homologs, this specific toxin family has been renamed the MARTX (multifunctional autoprocessing RTX) toxin [77]. 

Early characterizations of the MARTX toxin were mainly conducted using *V. cholerae* El Tor strain N16961, and thus the toxin was considered as an accessory toxin behind the highly potent CTX [73,74,81]. However, multiple independent studies conducted with other pathogenic *Vibrio* species, such as *V. vulnificus* and *V. anguillarum*, revealed that the MARTX toxin is essential for the pathogen’s cytotoxicity in vitro and pathogenicity in vivo [41,42,82,83,84]. Indeed, unlike the *V. cholerae* MARTX toxin, which has never shown hemolytic or cytolytic activity [73,74], a MARTX toxin from *V. vulnificus* exhibited rapid cytolytic activity by forming pore-like structures with an estimated radius of 1.63 nm on the target cell membrane. Consequently, the intoxicated host cells released their cytosolic contents, including lactate dehydrogenase, into culture supernatants [41,42,82]. Recently, the cytolytic activity of the *V. vulnificus* MARTX toxin has been linked to the repeats-containing regions at its amino- and carboxyl-terminal arms [85]. Consistent with its absence of cytolytic activity, the same regions of the *V. cholerae* MARTX toxin did not lyse the host cells [86].

**Figure 2:**
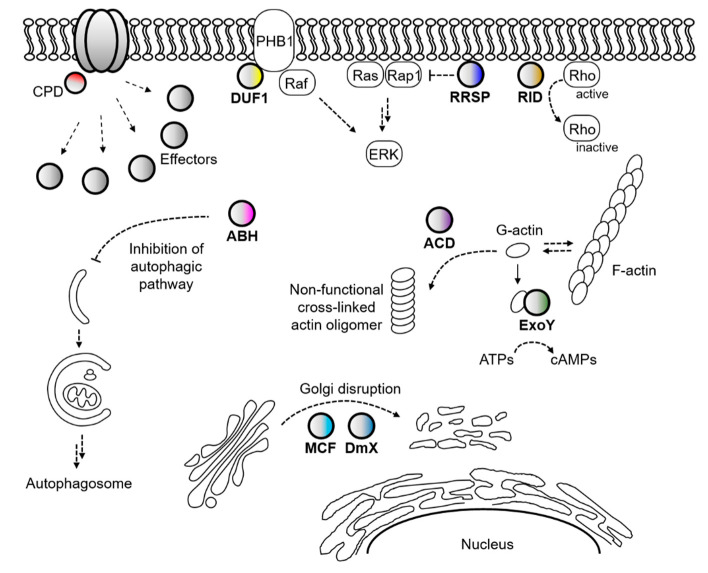
MARTX toxin effector domains and their effects on host cell targets. After release from the holo-MARTX toxin, effector domains interact with their target proteins or molecules in the host cell cytosol, exhibiting cytopathic consequences. PHB1, prohibitin-1; G-actin, monomeric globular actin; F-actin, fibrous actin.

Not only the actin-cross linking domain and Rho-inactivation domain but also many other cytopathic domains have been identified in *Vibrio* MARTX toxins (Figure 2) [78,80]. For instance, autophagy and endosomal trafficking of infected cells were inhibited by an alpha/beta hydrolase domain (ABH) of the *V. cholerae* MARTX toxin [87]. Prohibitin-1 was identified as an interacting partner of the domain of unknown function at the first position (DUF1) of the *V. vulnificus* MARTX toxin and suggested to serve as a receptor for the toxin [88]. The integrity of Golgi was affected by both the makes caterpillars floppy-like and domain X domains (MCF and DmX) [89,90,91], and Ras-ERK signaling was disrupted by the Ras/Rap1-specific endopeptidase domain (RRSP) of the *V. vulnificus* MARTX toxin [92,93,94]. In addition, cyclic AMPs were massively produced in intoxicated cells by an actin-stimulated ExoY-like domain [95,96].

Because varying numbers and combinations of these cytopathic effector domains are present in each different MARTX toxin, and they are delivered by the toxin itself, the MARTX toxin is somewhat similar to the type 3 secretion system (T3SS) or T4SS of bacterial pathogens [78,97]. However, the consequence of MARTX intoxication is more complicated because MARTX effectors are delivered into the target cell all at once [98,99]. Nonetheless, the MARTX effectors can be relatively easily coordinated with other virulence factors at the transcriptional level than the T3S or T4S effector proteins. This is because the MARTX effectors are residing in a single protein, the MARTX toxin, as modular domains.

### 2.3. Secreted Phospholipases

Phospholipases are lipolytic enzymes that hydrolyze ester or phosphoester linkages in phospholipids. Because the cell membranes of eukaryotes are mainly composed of phospholipid bilayers, phospholipases secreted by pathogens often act as virulence factors by affecting the integrity of the cell membrane. In addition, some phospholipases can elicit or dysregulate cell signaling pathways involved in apoptosis or inflammation because they eventually produce diverse signaling molecules such as arachidonic acid and lysophosphatidylcholine (LPC) [100,101]. For example, *Clostridium perfringens* produces cytotoxic phospholipase C, which causes chloride secretion from rat colons [102], and *Pseudomonas aeruginosa* produces and translocates the T3S effector ExoU, a patatin-like phospholipase A_2_ associated with lung injury and sepsis in mice [103]. 

Many *Vibrio* species also produce and secrete phospholipases into extracellular milieu [36,104,105,106,107,108]. In early studies, Mizuguchi and colleagues found a thermolabile hemolysin (TLH) gene from *V. parahaemolyticus* [109,110]. Later, the same group revealed that the TLH is actually a phospholipase with phospholipase A and lysophospholipase activity [111]. Since then, independent groups have reported several homologous proteins from various *Vibrio* species. Based on sequence homology, the TLH family of phospholipases includes the TLH of *V. parahaemolyticus*, Lec (lecithinase) of *V. cholerae*, PhlA (phospholipase A) of *V. mimicus*, VHH (*V. harveyi* hemolysin) of *V. harveyi*, *Va*PlpA (*V. anguillarum* phospholipase A) of *V. anguillarum*, and *Vv*PlpA (*V. vulnificus* phospholipase A) of *V. vulnificus* [112]. It should be noted that all these proteins contain a putative signal peptide sequence for T2SS at the amino-terminus, and *Vv*PlpA has actually been found to be secreted via that system [36,112]. Thus, TLH family phospholipases in vibrios are believed to act as exotoxins after being secreted from the bacterium (Figure 1c).

Despite the substantial level of sequence homology, the biochemical properties of the secreted phospholipases are somewhat distinct from one another. For instance, the TLH of *V. parahaemolyticus* can hydrolyze both fatty acid esters in phosphatidylcholine (PC) positioned at the sn-1 and sn-2 sites. It can also hydrolyze LPC, producing a free fatty acid and glycerophosphorylcholine [111]. In contrast, the *V. mimicus* PhlA does not possess lysophospholipase activity, even though it cleaves both sn-1 and sn-2 sites like TLH [106]. No lysophospholipase activity had been detected with *V. anguillarum Va*PlpA or *V. vulnificus Vv*PlpA, but a fatty acid at sn-2 site was preferentially cleaved, indicating that these two enzymes belong to the phospholipase A_2_ family [36,108]. 

The hemolytic activity and cytotoxicity of the secreted phospholipases and their effects on the overall virulence of *Vibrio* species are also quite different. PhlA, VHH, and *Va*PlpA have been shown to have hemolytic activity only against erythrocytes obtained from fish such as rainbow trout, tilapia, or salmon [106,107,108]. Rabbit, human, and sheep erythrocytes exhibited very little or no lysis upon PhlA or *Va*PlpA treatment [106,108]. In contrast, *Vv*PlpA showed substantial levels of hemolytic activity on human and horse erythrocytes but not on sheep erythrocytes [36]. These results suggest that each phospholipase could require a different PC level in the target membrane to lyse it because the PC content of the total erythrocyte phospholipids in fish, rabbits, humans, and sheep are quite different each other (about 58%, 34%, 17%, and 4%, respectively) [106,113].

The in vivo potencies of the secreted phospholipases also differ widely. The culture supernatant of wildtype (WT) *V. cholerae*, but not the Lec mutant strain, caused human intestinal cell (HT29/C1) loss. However, in ligated rabbit ileal loop infection experiments, the Lec mutant showed a level of fluid accumulation comparable with that from the WT strain, indicating that the phospholipase activity of Lec is not a major factor responsible for fluid accumulation [105]. PhlA and VHH exhibited significant cytotoxicity in CHSE-214 fish cells and flounder gill cells, respectively [106,107]. But only the VHH preparation induced flounder death after intraperitoneal (i.p.) infection [107]. The most profound difference was observed between *Va*PlpA and *Vv*PlpA. Although the *V. anguillarum* mutant defective for *Va*PlpA showed a two- to three-fold decrease in hemolytic activity on fish blood agar, its virulence did not differ statistically from that of the WT strain in fish infection experiments [108,114]. In contrast, the *V. vulnificus* mutant defective for *Vv*PlpA showed significantly attenuated virulence in mice infection experiments, demonstrating that *Vv*PlpA is a crucial virulence factor for this pathogen. *Vv*PlpA also contributed to the rapid necrotic death of human epithelial INT-407 cells [36].

Recently, the three-dimensional crystal structure of *Vv*PlpA was resolved at 1.4-Å resolution [112]. The protein consists of two close-packed domains, an amino-terminal domain of unknown function, and a carboxyl-terminal phospholipase domain. Although no function has yet been suggested for the amino-terminal domain, it is speculated to play important roles in the TLH family of secreted phospholipases because a central part of the domain, the β1 strand, is strictly conserved in all these phospholipases [112]. Because the *V. parahaemolyticus* TLH showed lecithin-dependent hemolytic activity and the other secreted phospholipases exhibited PC-dependency for erythrocyte lysis, it would be useful to determine whether the amino-terminal domain senses a PC molecule on host cell membranes and induces concomitant conformational changes in the carboxyl-terminal active domain. Another noticeable feature discovered by the structural study is that the catalytic site in *Vv*PlpA seems to be degenerated and consists of a Ser-His-Gly triad instead of a Ser-His-Asp/Glu triad. A chloride ion sitting at the catalytic site, however, functions as a substitute for the acidic Asp/Glu residue. Among the various TLH family of secreted phospholipases in *Vibrio* species, only *Vv*PlpA and *Va*PlpA, which show phospholipase A_2_ activity, have a Gly, not an Asp or Glu, at the third position of the catalytic triad. Therefore, it would also be intriguing to test whether this variation in the catalytic triad is related to the specific activity of the TLH family of secreted phospholipases.

## 3. Coordinated but Distinct Regulation of Exotoxin Genes in *Vibrio* Species

To determine the factor regulating the *V. cholerae* hemolysin/cytolysin gene *hlyA*, Williams and Manning screened a genomic library of the *V. cholerae* O17 strain in *E. coli* along with an *hlyA*-*cat* fusion reporter plasmid [115]. By characterizing the genomic library region of clones that showed increased chloramphenicol acetyl transferase (cat) activity, they first discovered a *hlyU* gene and characterized its product, HlyU, as an activator of the *hlyA* [115,116]. More than a decade later, a homologous protein in *V. vulnificus* was found to positively regulate both the *rtxA* and *plpA* genes, which encode the MARTX toxin and secreted phospholipase A_2_, respectively [36,42]. Similarly, HlyU in *V. anguillarum* has been found to regulate the expression of the *vah1*-*plp* and *rtxACH-BDE* clusters encoding cytolysin/hemolysin, secreted phospholipase, and MARTX toxin system [117]. The detailed mechanisms of this HlyU-mediated transcriptional regulation of major *Vibrio* exotoxins will be summarized and compared in the following sections after a short review of the HlyU protein itself.

### 3.1. HlyU, a Common Transcriptional Regulator of Major Exotoxin Genes in *Vibrio* Species

HlyU is a small, homo-dimeric transcriptional regulator widely distributed in many *Vibrio* species. Structural analyses of *V. vulnificus* and *V. cholerae* HlyUs revealed that the protein belongs to the ArsR/SmtB family of transcriptional regulators [118,119]. Like other members of this family, HlyU harbors a winged helix-turn-helix (wHTH) motif predicted to interact with the target DNA molecule. Generally, a recognition helix in the wHTH motif is inserted into a major groove, whereas the winged region composed of short antiparallel β-strands is inserted into a minor groove of the DNA [120]. Although the complex structure of DNA-bound HlyU is not yet available, that DNA binding mode has been repeatedly proposed by independent groups conducting structural modeling study [118,119,121]. All those binding models suggest that the DNA bends upon HlyU binding because the distance between the two recognition helices in the dimeric form of HlyU is shorter than 34 Å, the distance between two consecutive major grooves in B-DNA.

A feature that distinguishes HlyU from other ArsR/SmtB family proteins is that it does not bind to metal ions [118,119,122]. ArsR, a profound example of this family of transcription factors, directly senses heavy metal arsenate using a highly conserved cysteine residue in the so-called “metal binding box.” This binding induces conformational changes in ArsR that lead to protein dissociation from the operator DNA molecule [123]. Interestingly, the “ELCV(C/G)D” motif of the metal binding box is degenerated and replaced with “ELSVGE” in the *Vibrio* HlyU proteins. Moreover, other metal binding residues in the second metal binding site in the dimeric interface of ArsR/SmtB family proteins are also replaced by non-metal binding residues in the *Vibrio* HlyUs [121]. Therefore, it appears that HlyU is not a metal-sensing transcriptional regulator [118,119]. 

Instead, Chakrabarti and colleagues proposed that HlyU might sense oxygen levels in its local environment and regulate its regulon as a redox switch [119,122]. Indeed, one of two sole cysteine residues in the *V. cholerae* HlyU was modified by sulfenic acid in a crystal structure, whereas that oxidation was absent if the structure was solved under reducing conditions [119]. Molecular dynamic simulations further suggest that the predicted sensing role of the cysteine residues could be linked to DNA binding activity because the distance between the oxidizing cysteine and another cysteine residue exhibited a strong correlation with the DNA binding status [122]. Although more direct evidence should be collected, this model is of great interest because pathogenic *Vibrio* species face microaerobic or anaerobic conditions when they invade the host intestinal tract [124].

### 3.2. Transcriptional Regulation of Cytolysin/Hemolysin Genes in *Vibrio* Species

Consistent with the role of hemolysin in releasing iron-containing proteins from erythrocytes, hemolysin synthesis was found to be iron-regulated in *V. cholerae* [125]. Furthermore, the introduction of the *E. coli fur* gene into the *V. cholerae* mutant strain that constitutively produces hemolysin reestablished the iron-dependent regulation of hemolysin synthesis. These results suggest that the transcription factor Fur, with iron as a corepressor, represses the cytolysin/hemolysin gene *hlyA* in *V. cholerae*, just as it does in *E. coli* [125]. HlyU was also found to positively regulate the transcription of *hlyA* [116]. Tsou and Zhu discovered that a quorum sensing master regulator, HapR, directly represses *hlyA* expression [28].

**Figure 3:**
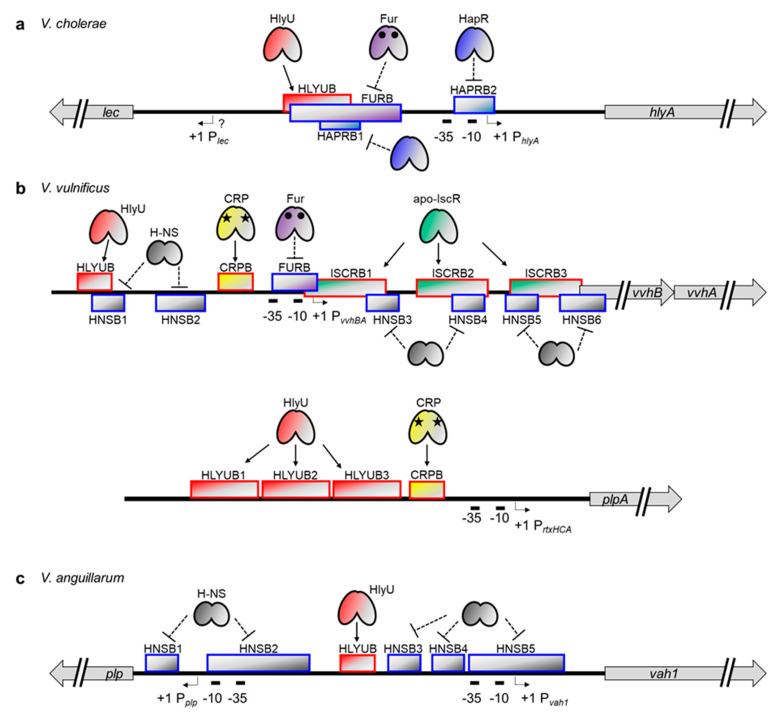
Transcriptional regulation of cytolysin/hemolysin and the secreted phospholipase genes in (**a**) *V. cholerae*, (**b**) *V. vulnificus*, and (**c**) *V. anguillarum*. Note that the phospholipase gene is divergently transcribed from the cytolysin/hemolysin gene in *V. cholerae* and *V. anguillarum*, but not in *V. vulnificus*. Transcription start sites and −10/−35 boxes are indicated by bent arrows and thick short lines, respectively. Solid arrows and dotted blunt lines represent the binding of positive and negative regulators, respectively. Binding sites for the positive and negative regulators are indicated by red and blue framed boxes, respectively. Black circles in Fur are irons, whereas black stars in CRP are cAMPs. HLYUB, HlyU binding site; FURB, Fur binding site; HAPRB1, HapR binding site 1; and so on.

Recently, precise binding sites were determined for Fur, HlyU, and HapR in the *hlyA* promoter of *V. cholerae* (Figure 3a) [43]. HlyU binds to the far upstream region of the *hlyA* promoter, from −195 to −131 bp relative to the transcription start site of *hlyA*, and activates the transcription. Although the exact mechanism of HlyU-mediated activation is not yet clear in *V. cholerae*, it might function as an anti-repressor, as shown for the *vvhA* and *rtxA* genes in *V. vulnificus* (see below) [32,35]. In contrast to HlyU, Fur represses *hlyA* by binding to a region spanning −164 bp to −75 bp. Notably, this site is partially overlapped with the HlyU binding site described above, suggesting that Fur binding could exclude the important positive regulator HlyU from the *hlyA* promoter, thereby enacting negative regulation. In the case of HapR, binding occurs at two different regions: from −158 bp to −119 bp and −31 bp to +3 bp. The first HapR binding site is, again, partially overlapped with the HlyU binding site, whereas the second site is overlapped with an RNA polymerase (RNAP)-binding site, namely −10 box and extended −10 region. The occupancy of these regions by HapR probably inhibits the binding of HlyU and RNAP and thus decreases the *hlyA* transcript level.

The transcriptional regulation mechanism of *vvhA*, a cytolysin/hemolysin gene in *V. vulnificus*, is somewhat different from and more complicated than that of the *hlyA* in *V. cholerae*. Most important, *vvhA* is co-transcribed with the upstream *vvhB* gene [31]. Also, a nucleoid-like protein, H-NS, has been found to bind to multiple sites in the *vvhBA* operator region to silence their expression (Figure 3b) [32,126]. Unlike HapR binding in the *hlyA* promoter, SmcR, a quorum sensing master regulator in *V. vulnificus*, does not bind directly to the *vvhBA* promoter. Instead, it indirectly regulates *vvhBA* expression by binding to the *hlyU* promoter and repressing its transcription [127].

Nonetheless, regulation mechanisms similar to those found in *hlyA* regulation do exist in *vvhBA* regulation. For example, *V. vulnificus* HlyU binds directly to the upstream promoter region of *vvhBA*. This HlyU binding site, spanning from −128 bp to −114 bp relative to the transcription start site of *vvhBA,* partially overlaps with one of the H-NS binding sites described above (Figure 3b). Therefore, HlyU binding could cause de-repression of *vvhBA* [32,127]. The *V. vulnificus* Fur protein also binds directly to the promoter region of *vvhBA* and represses its transcription [128,129]. In this case, however, the binding site spans from −32 bp to +2 bp, indicating that Fur binding directly hinders RNAP recruitment, not HlyU binding, to the promoter. It is also worth noting that the location of this Fur binding site is almost the same as the location of the second HapR binding site in the *V. cholerae hlyA* promoter (Figure 3a,b), suggesting that the prevention of RNAP binding via any repressor protein might be a common strategy for hemolysin gene regulation in *Vibrio* species [43,128]. 

In addition to Fur, HlyU, and H-NS, two global regulators are also involved in *vvhBA* regulation in *V. vulnificus*. First, a CRP protein, when complexed with a cyclic AMP, binds directly to the promoter region from −67 to −50 bp and activates *vvhBA* expression as a Class I activator (Figure 3b) [31,32,130]. Second, an IscR protein, a Fe-S cluster-containing transcriptional regulator, binds directly to three different sites in the *vvhBA* operator [32]. Intriguingly, all these IscR binding sites are located downstream of the *vvhBA* transcriptional start site. Nonetheless, these sites are overlapped with the H-NS binding sites, and thus IscR functions as an anti-repressor and enhances *vvhBA* transcription (Figure 3b) [32].

Relatively little information is available about the transcriptional regulation mechanism of *vah1* in *V. anguillarum*. Nonetheless, both H-NS and HlyU have been revealed to affect *vah1* expression by directly binding to the DNA [33,117]. Indeed, H-NS binds to multiple regions of the *vah1* promoter, including RNAP binding sites, -10 / -35 boxes, and represses *vah1* expression [117]. In contrast, HlyU binds to a single region and functions as a positive regulator [33]. Interestingly, however, the HlyU binding site does not overlap with any of the H-NS binding sites in the *vah1* regulation system (Figure 3c). Perhaps another transcriptional regulator recruited by HlyU functions as an anti-repressor by competing with H-NS for DNA binding and interferes with the H-NS-mediated silencing. It also cannot be ruled out that HlyU binding might hinder the cooperative binding of H-NS molecules on the DNA.

### 3.3. Transcriptional Regulation of the MARTX Toxin Genes in *Vibrio* Species

**Figure 4:**
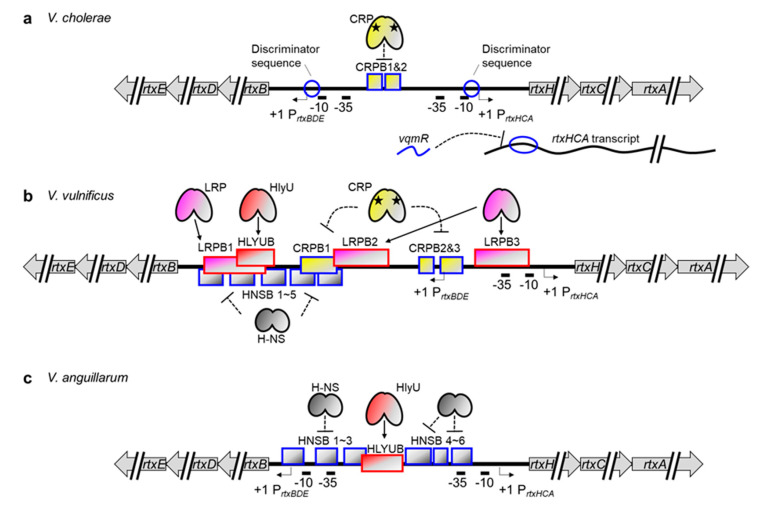
Transcriptional regulation of the MARTX toxin and its secretion system genes in (**a**) *V. cholerae*, (**b**) *V. vulnificus*, and (**c**) *V. anguillarum*. Note that the toxin encoding operon *rtxHCA* and the secretion system encoding operon *rtxBDE* are divergently transcribed. Discriminator sequences in the *V. cholerae rtxHCA* and *rtxBDE* are indicated by blue circles. A *vqmR* binding site in the *V. cholerae rtxHCA* transcript is indicated by a blue oval. The others are the same as described in Figure 3.

In *Vibrio* species, the MARTX toxin gene is in the *rtx* locus, which consists of two divergently transcribed operons [131,132]. One is *rtxHCA*, which encodes a conserved hypothetical protein, toxin-activating acyltransferase, and the MARTX toxin. The other operon is *rtxBDE*, which encodes atypical T1SS components for MARTX toxin secretion. Boardman and Satchell investigated the growth phase regulation of MARTX toxin activity in *V. cholerae* and discovered that both operons are highly expressed at the exponential phase but not the stationary phase [29]. Although they found no trans-acting regulators involved in this regulation, they successfully presented that a discriminator region downstream of the -10 box of *rtxBDE* is responsible for the growth phase–dependent regulation of the operon. Because a similar discriminator sequence is also present at the *V. cholerae rtxHCA* promoter, they further suggested that such a stringent sensing element could regulate the transcription of the MARTX toxin gene as well (Figure 4a) [29,133].

The transcriptional regulator proteins for the MARTX toxin gene were first identified in *V. vulnificus*. Because the *V. vulnificus hlyU* gene was preferentially upregulated in in vivo conditions and was critical for pathogenesis [134], Crosa and colleagues hypothesized that genes encoding crucial virulence factors were controlled by this regulator. In a microarray analysis comparing WT and *hlyU* mutant *V. vulnificus*, they found decreased expression of *rtxHCA* in the mutant. Consistent with that, a recombinant HlyU protein bound directly to the promoter region of *rtxHCA* (Figure 4b) [42]. They further discovered that H-NS also binds to multiple regions of the promoter and silences the operon. Notably, HlyU binding alleviates this H-NS-mediated repression, as in the *vvhBA* promoter [32,35].

Although little information is available, MARTX toxin gene regulation in *V. anguillarum* appears to be similar to that in *V. vulnificus.* The *V. anguillarum* H-NS binds to six sites in the promoter region of the *V. anguillarum rtxHCA* operon, including the -35 box, and represses transcription (Figure 4c). One of the six sites, however, overlaps with the HlyU binding site, and thus HlyU can function as an anti-repressor of the *V. anguillarum rtxHCA* promoter [33,117].

Recently, additional regulation mechanisms for the MARTX toxin gene have been revealed in both *V. cholerae* and *V. vulnificus*. First, while trying to determine the quorum-controlled small regulatory RNAs in *V. cholerae*, Papenfort and colleagues discovered a *vqmR* that represses *rtxHCA* expression by binding to the 5′ UTR of the transcript (Figure 4a) [135,136]. Although this post-transcriptional regulation is not conserved in *V. vulnificus* [135], quorum-mediated repression of *rtxHCA* could still be operating in that strain because the quorum sensing regulator SmcR directly represses *hlyU,* whose product alleviates the H-NS-mediated repression of the *rtxHCA* [127]. 

Second, the leucine-responsive regulatory protein (LRP) and CRP have been found to control *rtxHCA* in *V. vulnificus* via direct binding (Figure 4b) [137]. Despite the partial overlap between the LRP and H-NS binding sites, both proteins can bind simultaneously to the *rtxHCA* promoter. In fact, LRP-mediated activation of *rtxHCA* occurred independently of H-NS, indicating that LRP is an authentic activator, not an anti-repressor like HlyU, for the *V. vulnificus* MARTX toxin gene. It has also been found that the cAMP-complexed CRP protein binds to three different upstream regions of the transcription start site of *rtxHCA* and negatively regulates transcription [137]. Those authors speculated that this could be due to a hindered promoter-clearance of RNAP because CRP retains RNAP via protein–protein interaction. This rather unusual regulation by CRP is not unique to this particular case but has also been reported in the *V. cholerae rtxBDE* promoter (Figure 4a) [138]. Notably, however, the *V. cholerae rtxHCA* operon was constitutively transcribed independently of CRP [138], suggesting that the CRP-mediated regulation of the *rtx* genes is similar but not strictly conserved among different *Vibrio* species.

Another thing should be noticed here is that the transcription start site of the *V. vulnificus rtxBDE* is located at the third CRP binding site (CRPB3) (Figure 4b) [132]. Thus, most of the LRP, CRP, and HlyU binding sites are downstream of the transcription start site of *rtxBDE*. Nonetheless, the roles of those transcriptional regulators on *rtxBDE* expression have not yet been elucidated. For a comprehensive understanding of the regulation of MARTX toxin secretion, it also needs be determined whether those regulators function as roadblocks or anti-repressors for *rtxBDE* expression.

### 3.4. Transcriptional Regulation of Secreted Phospholipase Genes in *Vibrio* Species

In contrast to the two exotoxins just described, the transcriptional regulation of secreted phospholipases has not been studied in detail. In *V. cholerae* and *V. anguillarum*, the secreted phospholipase genes (*lec* and *plp*, respectively) are divergently transcribed from the cytolysin/hemolysin genes (*hlyA* and *vah1*, respectively), suggesting that two exotoxin genes could share a regulatory region and thus be controlled by a common transcriptional regulation mechanism (Figure 3a,c). Indeed, it was reported that the expression of the *plp* gene in *V. anguillarum* is directly repressed by H-NS and that repression is suppressed by HlyU binding, like the *vah1* gene [33,117]. Although no such definitive study has been done, the same gene synteny for *lec* and *hlyA* in *V. cholerae* strongly suggest that the *lec* gene is similarly regulated with *hlyA* by HlyU and other repressors such as Fur and HapR [43,105,114].

In *V. vulnificus*, however, the phospholipase gene *plpA* is not physically linked to cytolysin/hemolysin gene *vvhBA* and is located at an entirely different site on the chromosome with its own regulatory region (Figure 3b). Despite that unique gene structure, the *V. vulnificus plpA* is also under the control of HlyU [36]. Indeed, HlyU binding occurs at three consecutive sites (from −191 bp to −157 bp, −151 bp to −128 bp, and −126 bp to −93 bp relative to the transcription start site of *plpA*) in the region upstream of the *plpA* promoter. However, no evidence for H-NS binding has been found, indicating that HlyU, in this case, may function as an activator not an anti-repressor. It has also been found that the CRP protein binds to the region from −82 bp to −57 bp and executes positive regulation as a Class I activator [36]. Notably, no regulation hierarchy or competition has been found between HlyU and CRP. Instead, they activate the *plpA* gene in an additive manner.

### 3.5. Spatiotemporal Regulation of Exotoxin Genes for Successful Pathogenesis

As summarized above, diverse transcriptional regulators cooperate to control three major exotoxin genes in pathogenic *Vibrio* species. These include HlyU, H-NS, Fur, HapR (or its homologues like SmcR), CRP, IscR, and LRP. The small regulatory RNA *vqmR* and discriminator sequences in the promoters have also been found in the regulation system. 

**Figure 5:**
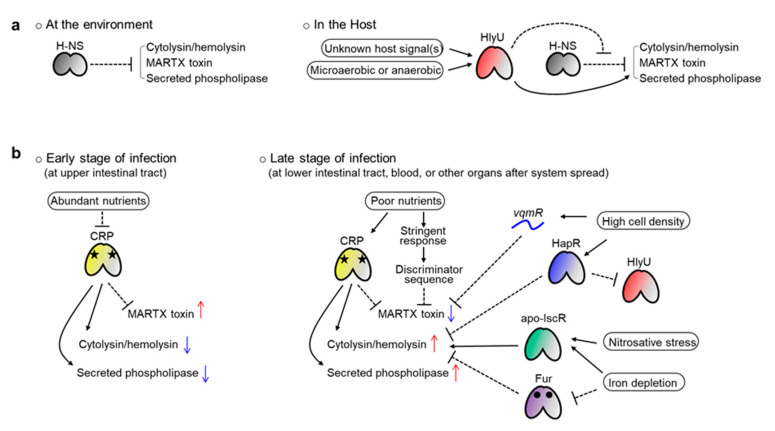
Spatiotemporal regulation of the *Vibrio* exotoxin genes by diverse transcriptional regulators. (**a**) In a non-host environment, exotoxin gene expression is repressed by H-NS. When *Vibrio* encounters a host, unknown host signal(s) induce HlyU production, de-repressing the H-NS-mediated repression of exotoxins. HlyU might also sense microaerobic or anaerobic conditions in the host. *V. cholerae* cytolysin/hemolysin and *V. vulnificus* secreted phospholipase expression are also activated by HlyU. (**b**) Exotoxins are differentially regulated depending on the local host conditions. At the early stage of infection, abundant nutrients inactivate CRP, increasing the MARTX toxin but decreasing cytolysin/hemolysin and secreted phospholipase. At the late stage of infection, poor nutrient conditions are conveyed by CRP and discriminator sequences, decreasing the MARTX toxin but increasing cytolysin/hemolysin and secreted phospholipase. Nitrosative stress and iron depletion are sensed by IscR and Fur, resulting in the production of cytolysin/hemolysin. When the pathogen overwhelms the host at the very late stage of infection, the quorum sensing master regulator HapR and quorum-regulated sRNA *vqmR* repress the expression of MARTX toxin and cytolysin/hemolysin, respectively. HapR (or its homologue, SmcR) also represses HlyU, terminating the HlyU-mediated de-repression of exotoxin genes.

Why do pathogens use these diverse factors in exotoxin production? First, because the genes should be expressed preferentially when *Vibrio* invades the host. The major role of all three exotoxins is attacking host cells or tissues and using their destructive activities to dampen host defense systems. Therefore, toxin production is not required if *Vibrio* lives freely in the ocean, and needless production would squander energy. This is particularly critical for MARTX toxin production because this toxin is usually encoded by the longest gene of *Vibrio* species [78]. Thus, the nucleoid-like protein H-NS is used in most of these exotoxin gene promoters to tightly repress them (Figure 5a) [32,117,127,137]. 

When *Vibrio* encounters a host, however, this H-NS-mediated silencing should be alleviated, and HlyU acts at that stage as an anti-repressor (Figure 5a). Indeed, HlyU has a higher affinity to the target DNA compared with H-NS, and thus can outcompete the H-NS [32,35,117]. Notably, in some promoters, like the *V. cholerae hlyA* and *V. vulnificus plpA*, where no H-NS binding has been found, HlyU also acts as an activator [36,43]. These results confer a critical role on HlyU, over H-NS, as a key virulence regulator in pathogenic *Vibrio* species. Because *hlyU* is preferentially expressed in in vivo conditions [134], all three exotoxins can escape H-NS-mediated silencing when *Vibrio* invades a host. Then, the genes would be ready for the next round of regulation conducted by their own distinct transcriptional regulators.

Second, even if *Vibrio* has entered a host, each exotoxin must be expressed at the right time and in the right place because each exotoxin has a distinct function. The MARTX toxin dampens immune-related signaling in host cells and damages protective barriers in the host gut [78,80]. Meanwhile, cytolysin/hemolysin lyses erythrocytes to liberate iron-containing proteins and induces destructive inflammation by activating cytokine production from immune cells [47,48,71,72]. Similarly, secreted phospholipases lyse host cells, facilitating necrotic cell death and subsequent inflammation [36]. Given those functions, the MARTX toxin should be expressed from the early stage of infection, when *Vibrio* encounters the host gut epithelium. In contrast, cytolysin/hemolysin and phospholipase are necessary at a relatively later stage of infection, when *Vibrio* has overcome the host’s initial immune responses and started to bloom to overwhelm the host. 

Because local conditions differ spatiotemporally in the host, *Vibrio* should sense those differences to control exotoxin expression. It is in this context that the role of the diverse transcriptional regulators can be explained. For example, CRP can regulate genes depending on local nutrient levels. Relatively more nutrients would be present in the host’s upper intestinal tract (duodenum and jejunum) than in lower sites (ileum). When *Vibrio* reaches the upper site after ingestion with contaminated foods, those relatively abundant nutrients, such as glucose, could lead the depletion of cAMP in the bacterium. Consequently, cAMP-unbound, inactivated CRPs would be released from the *rtxHCA* and *rtxBDE* promoters, allowing the expression and secretion of the MARTX toxin (Figure 5b) [137,138]. Conversely, toxin expression would be repressed by cAMP-bound CRPs when *Vibrio* encounters nutrient-poor conditions in the lower intestinal tract or at the late stage of infection. The discriminator sequences found in the *V. cholerae rtx* operons might also contribute to this kind of nutrient level–dependent regulation of the MARTX toxin. In contrast to the *rtxHCA*, both the *vvhBA* and *plpA* genes are activated by CRP in *V. vulnificus* [32,36]. Therefore, those two exotoxins could be expressed at the late stage of infection, when nutrients are relatively depleted (Figure 5b). 

Other examples are IscR and Fur. If *Vibrio* species, especially *V. vulnificus*, invade a host’s blood system, the pathogens will face harsh conditions, such as iron depletion and nitrosative stress, due to the host’s iron-sequestering system and NO-producing immune cells (macrophages and neutrophils) [139]. Because many more apo-IscRs (IscR with no Fe-S cluster) are produced under iron starvation and nitrosative stress conditions, the *vvhBA* regulatory region will be occupied by apo-IscR instead of H-NS in such conditions [32,140]. In that way, the H-NS-mediated silencing of *vvhBA* can be alleviated, allowing the *Vibrio* to overcome the stresses and even counterattack the host using cytolysin/hemolysin (Figure 5b). Similarly, the Fur-mediated repression of the *V. vulnificus vvhBA* and *V. cholerae hlyA* will be relieved under iron depletion conditions because apo-Fur (Fur without iron) cannot bind to the target DNAs (Figure 5b) [32,128].

Finally, quorum sensing also contributes to the precise regulation of exotoxin expression. If the invading pathogens successfully colonize and overwhelm the host, their population will expand significantly. It is tempting to speculate that no more energy-consuming and destructive exotoxins such as MARTX might be necessary at that very late stage of infection. In this context, both HapR- and *vqmR*-mediated repression of *V. cholerae hlyA* and *rtxHCA*, respectively, are significant at a high bacterial cell density [43,135]. Furthermore, because the *hlyU* genes in *V. cholerae* and *V. vulnificus* are directly repressed by HapR and SmcR, respectively, all three exotoxins could be controlled by quorum signals in a similar way (Figure 5b) [43,127].

## 4. Anti-Virulence Strategy Targeting HlyU and Future Directions

Considering the increasing threat of antibiotic resistance, alternative strategies to combat bacterial pathogens urgently need to be developed [141]. This is also true of pathogenic *Vibrio* species because a tremendous amount of antibiotics is currently used in aquaculture worldwide [142,143]. One alternative is an anti-virulence strategy that targets bacterial virulence potential instead of viability [144,145]. Indeed, a treatment that inhibited the expression, secretion, or activity of virulence factors such as exotoxins could disarm the pathogen and facilitate pathogen elimination from the host. 

As reviewed in this article, the key virulence regulator HlyU coordinately controls three major exotoxins in pathogenic *Vibrio* species. Although not summarized here, it also regulates T3SS1 genes in *V. parahaemolyticus* via the transcription factor ExsA [146]. Therefore, HlyU is a fascinating target for developing anti-virulence agents to impede the cooperative consequences of the exotoxins in pathogenic *Vibrio* species. Compared with the WT *V. vulnificus*, a *hlyU* deletion mutant indeed showed a 10- to 50-fold increase in LD_50_ in a mouse infection model [134]. Because HlyU binds directly to the toxin gene promoters and positively regulates them as an anti-repressor or activator, inhibiting the DNA binding activity of HlyU could reduce virulence. 

Recently, three small molecules have been identified as HlyU inhibitors [147,148,149]. Although they have no structural similarity, both *N*-(4-oxo-4H-thieno[3,4-c]chromen-3-yl)-3phenylprop-2-ynamide (CM14) and 2′,4′-dihydroxychalcone interfere in the binding between HlyU and the *V. vulnificus rtxA* gene promoter thus decrease MARTX toxin expression [148,149]. No such DNA binding defect has been shown in the fursultiamine hydrochloride–treated HlyU, but the expression of the *rtxA* gene was also significantly affected in that case [147]. Consistent with HlyU’s global regulatory function in *Vibrio* species, these HlyU inhibitors, especially CM14, exhibited strong anti-virulence effects against various pathogenic *Vibrio* species by inhibiting expression of multiple exotoxin genes [148,149].

Moving forward, researchers need to keep pursuing a comprehensive understanding of the virulence factors and their regulation in pathogenic *Vibrio* species to establish more effective and practical anti-virulence strategies. First, the characteristics and regulation mechanisms of the three exotoxins still need to be clearly identified. What mediates host cell binding of the MARTX toxin and secreted phospholipases? Why and how does the *V. vulnificus* MARTX toxin, but not the *V. cholerae* MARTX toxin, lyse host cells? Is it because of different expression levels caused by distinct regulation? Is the expression of *rtxHCA* in *V. cholerae* and other *Vibrio* species also controlled by nutrient availability via CRP or LRP, as found in *V. vulnificus*?

Second, HlyU-mediated virulence regulation should be further examined. Are any other exotoxins directly regulated by HlyU? How is their regulation coordinated with that of the three exotoxins reviewed here? Does HlyU really sense in vivo oxygen levels to regulate its regulon? Researchers also need to identify the conditions that cause HlyU to be preferentially expressed in vivo, initiating a virulence cascade.

Third, the exact modes of action of the HlyU inhibitors need to be revealed. Although it was identified that part of CM14 is covalently attached to a cysteine residue of HlyU, neither the initial interaction between CM14 and HlyU nor the binding mode of CM14 to HlyU has been clearly shown at the atomic level with its three dimensional structure [149]. That information would enable us to design a better HlyU inhibitor with improved activity and bioavailability compared with current treatments.

Last, not only the HlyU inhibitor but also inhibitors targeting other transcriptional regulators or the exotoxins themselves should be screened and identified. For example, researchers could screen for a small molecule that prevents iron sensing by Fur or nitrosative stress sensing by IscR. Similarly, the diverse quorum sensing inhibitors already found in multiple *Vibrio* species could be combined with those inhibitors to dysregulate the entire exotoxin gene expression system [150,151]. In addition, molecules that interfere in either the binding of cytolysin/hemolysin to the host cell membrane or the translocation of the MARTX effector domains into host cell cytosol could be combined. Because both spatiotemporal expression and the precise activity of multiple exotoxins are critical for successful pathogenesis of *Vibrio* species, such combinations could offer more-effective virulence attenuation than a HlyU inhibitor alone.

In conclusion, the functions and transcriptional regulation mechanisms of cytolysin/hemolysin, the MARTX toxin, and secreted phospholipases in pathogenic *Vibrio* species have been reviewed here. Notably, the transcriptional regulator HlyU coordinates the expression of all three exotoxins as an anti-repressor or activator. Despite this HlyU-mediated coordination, however, the expression of the exotoxin genes remains distinct. This is because diverse transcriptional regulators sense various host-driven environmental changes and then precisely regulate each exotoxin gene in a spatiotemporal manner. Along with the results from the research suggested above, this information will enable us to fight against pathogenic *Vibrio* species in the upcoming post-antibiotic era. Moreover, the strategy disrupting a coordinated but distinct regulation system of exotoxins could be applied to other pathogenic bacteria.
